# Arctic cyclone water vapor isotopes support past sea ice retreat recorded in Greenland ice

**DOI:** 10.1038/srep10295

**Published:** 2015-05-29

**Authors:** Eric S. Klein, J. E. Cherry, J. Young, D. Noone, A. J. Leffler, J. M. Welker

**Affiliations:** 1 University of Alaska Anchorage, Department of Biological Sciences; 2 University of Alaska Fairbanks, International Arctic Research Center; 3 Oregon State University, College of Earth, Ocean and Atmospheric Sciences; 4 South Dakota State University, Natural Resources Management

## Abstract

Rapid Arctic warming is associated with important water cycle changes: sea ice loss, increasing atmospheric humidity, permafrost thaw, and water-induced ecosystem changes. Understanding these complex modern processes is critical to interpreting past hydrologic changes preserved in paleoclimate records and predicting future Arctic changes. Cyclones are a prevalent Arctic feature and water vapor isotope ratios during these events provide insights into modern hydrologic processes that help explain past changes to the Arctic water cycle. Here we present continuous measurements of water vapor isotope ratios (δ^18^O, δ^2^H, *d-excess*) in Arctic Alaska from a 2013 cyclone. This cyclone resulted in a sharp *d-excess* decrease and disproportional δ^18^O enrichment, indicative of a higher humidity open Arctic Ocean water vapor source. Past transitions to warmer climates inferred from Greenland ice core records also reveal sharp decreases in *d-excess*, hypothesized to represent reduced sea ice extent and an increase in oceanic moisture source to Greenland Ice Sheet precipitation. Thus, measurements of water vapor isotope ratios during an Arctic cyclone provide a critical processed-based explanation, and the first direct confirmation, of relationships previously assumed to govern water isotope ratios during sea ice retreat and increased input of northern ocean moisture into the Arctic water cycle.

Dramatically shrinking perennial sea ice^1^ is changing Arctic hydrology, with impacts to both marine and terrestrial systems^2^,^3^,^4^. Declining sea ice extent is attributed to a general increase in global and Arctic temperatures caused by rising CO_2_ concentrations^2^,^5^. Arctic sea ice extent and warming temperatures are of great interest because retreating sea ice decreases the Earth’s albedo, which leads to further reduction in Arctic surface reflectivity and a positive feedback to the global thermal balance^6^. Moreover, declining perennial sea ice extent can influence the intensity of storms, as greater open water in the Arctic Ocean can lead to increased wind speed^7^. Arctic cyclones, especially during summer, are more common than previously believed^8^ and could be increasing in frequency in association with sea ice decline and warming in the Arctic^9^,^10^,^11^,^12^,^13^. These intense storm events are especially important as their strong winds can fracture sea ice, particularly in the summer when sea ice coverage is already diminishing^14^, which increases open water and mixing in the Arctic Ocean boundary layer^1^,^12^.

These sea ice reductions can also influence Arctic hydrology and water distribution, including increased precipitation linked to open water in the Arctic Ocean^15^. As the isotope ratios of water vapor and precipitation at high latitudes are collective reflections of moisture sources and temperatures^16^, they reveal mechanistic information about the processes influencing the Arctic water cycle. In July of 2013, a low pressure system developed over northern Scandinavia and intensified into a cyclone as it tracked eastward toward northern Alaska. This cyclone lasted approximately five days (July 23^rd^-July 27^th^) and had a minimum core pressure of ~978 hPa^17^. Fig. 1 shows the signature of this Arctic cyclone recorded at Toolik Lake Field Station (hereafter Toolik) in the Alaskan Arctic. Toolik (68° 38’ N, 149° 36’ W) is in the northern foothills of the Brooks Range, about 185 km south of the Arctic Ocean, at an elevation of about 760 m (Fig. 2 and Fig. S3). Although this cyclone was analyzed because it was large enough to bring moisture inland to Toolik, its isotope characteristics are likely representative of Arctic cyclones in general. This cyclone provided an important research opportunity to use the isotope ratios of water vapor to study how different moisture sources with varying humidity levels and sea ice conditions can influence Arctic water isotope ratios.

As water isotope ratios of modern hydrologic processes can help explain isotopic variability in paleoclimate records^18^,^19^,^20^, continuous measurements of modern water vapor isotope ratios associated with altered sea ice and ocean conditions are essential to understanding how past climate changes influenced isotopes of the Arctic water cycle. Specifically, Greenland ice core records suggest an isotopically distinct decoupling, or separation, between cooler temperatures at oceanic moisture sources and warmer temperatures at ice sheet depositional sites during past warming periods going back more than 100,000 years^21^,^22^. It is hypothesized that these isotope and associated temperature differences during past warming periods were caused by sea ice retreat and subsequent exposure of cool, open ocean water, which contributed moisture to the isotope signal deposited on the Greenland Ice Sheet^23^. Therefore, an understanding of processes controlling isotope ratios in water vapor during a recent increase in open water and atmospheric humidity, as may be found in an unusually intense, warm-season Arctic cyclone, provides context to help explain the water isotope ratios during past periods of Arctic sea ice retreat preserved in paleoclimate ice core records.

## Water isotope ratio characteristics, moisture sources, and *d-excess* values

**Figure 1:**
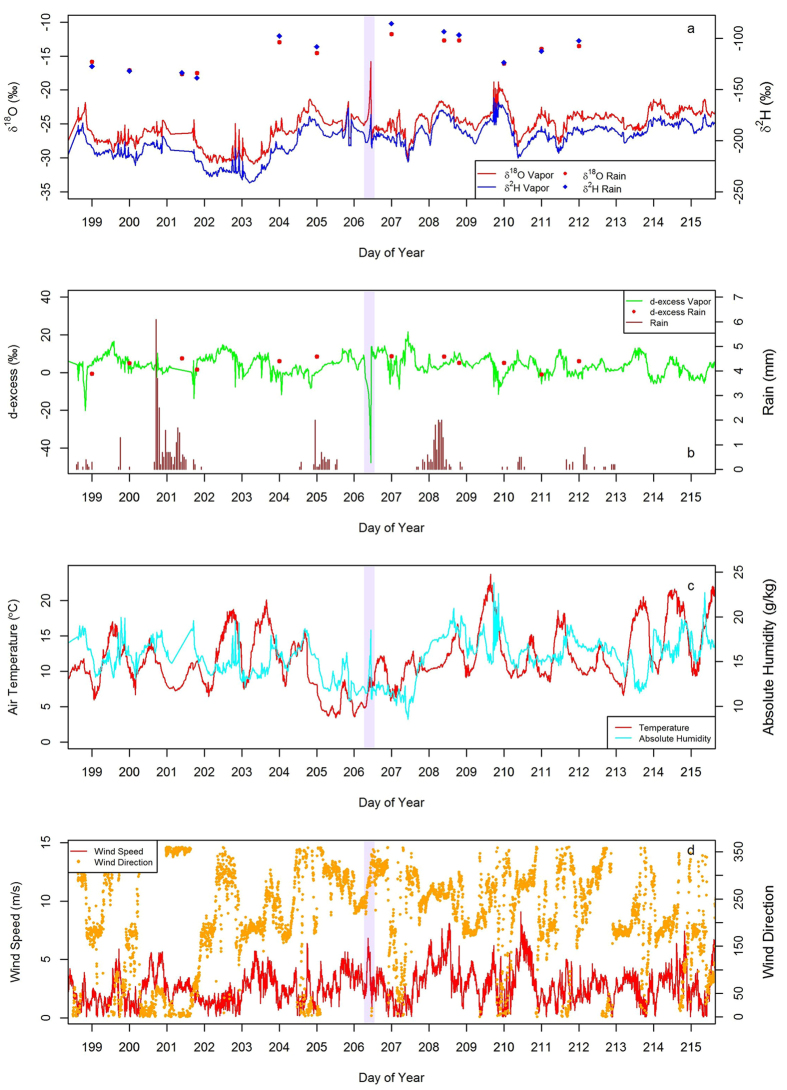
Isotope and weather data from Toolik Lake, AK (five minute averages) for the time period before and after an Arctic cyclone event (highlighted vertical bar). Large excursions in vapor δ^18^O (Panel a) and *d-excess* (Panel b) are apparent during the cyclone event. There were spikes in absolute humidity (Panel c) and wind speed (Panel d) that coincided with the isotopic excursions. The wind direction during the cyclone isotope excursion (~290°) is consistent with the directional movement of the cyclone (Fig. S4a).

Water vapor isotope ratio values during the cyclone event were different from values before and after the cyclone, as recorded at Toolik. The average δ^18^O, δ^2^H, and *d-excess* values during the cyclone event were −17.6‰, −181.0‰, and −39.4‰, respectively (Fig. 1 and Table S1). This compares to average values over the two week period (one week prior and one week after) across the cyclone event of −25.6‰, −200.4‰, and 4.31‰ for δ^18^O, δ^2^H, and *d-excess*, respectively. Further, the overall average values from late spring to late summer (i.e., the complete study period) for δ^18^O, δ^2^H, and *d-excess* were −28.2‰, −222.8‰, and 2.96‰. The lowest *d-excess* value (−47.8‰) and the most enriched δ^18^O value (−15.8‰) across the course of the entire record happened during the cyclone event (Table S1 and Fig. S2). Additionally, these are the lowest and most enriched *d-excess* and δ^18^O, respectively, Arctic water vapor isotope values ever measured^24^,^25^.

**Figure 2:**
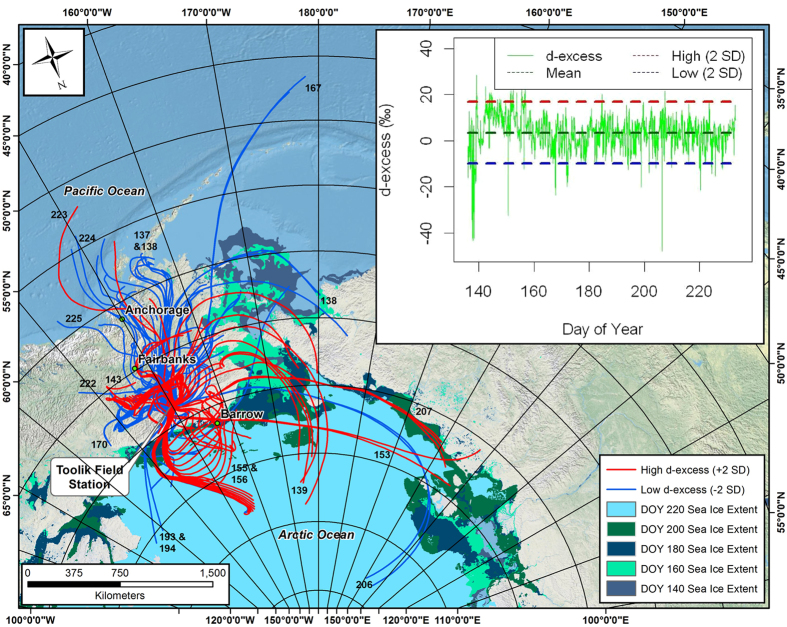
Air parcel back trajectories (main) and high and low *d-excess* values (inset). The time (Day of Year) associated with selected trajectories are presented in black numbers. Over 90% of high *d-excess* values (depicted with red lines) did not cross open water sources and generally had more northern trajectories, while nearly 90% of low *d-excess* values (depicted with blue lines) had trajectories crossing open waters and generally more southern trajectories. Figure created using ArcGIS 10.1.

Air parcel back trajectories were estimated and connected with both high and low groups of *d-excess* values, with high values greater than two standard deviations above the mean *d-excess* value and low values greater than two standard deviations below the mean *d-excess* value. This relationship indicates that the majority of high *d-excess* values had more northern trajectories, while the majority of low *d-excess* values had more southern trajectories (Fig. 2). This pattern of higher *d-excess* values associated with air parcels of Arctic origins and lower values related to air parcels with southern origins is consistent with water vapor *d-excess* values in NE Russia^24^ and Greenland^25^. Additionally, over 90% of high *d-excess* values had arid (i.e., no open water) vapor sources, while nearly 90% of low *d-excess* values had humid (i.e., open water) vapor sources. This is consistent with recent studies that found *d-excess* was negatively correlated with moisture source relative humidity^26^,^27^,^28^. Disproportionate enrichment in δ^2^H (relative to δ^18^O) indicates arid vapor sources and higher *d-excess* values while greater δ^18^O enrichment (relative to δ^2^H) indicates humid vapor sources and lower *d-excess* values^29^. Therefore, the high and low *d-excess* values in our data are consistent with expectations and reveal important and previously undocumented Arctic hydrological patterns that are essential to understanding the isotopic characteristics of the cyclone. These patterns indicate that a decrease in *d-excess* values in Arctic moisture is related to both the availability of open water at the vapor source and increased humidity. These characteristics appear to imprint on *d-excess* values, as shifts in the composition of oceanic surface waters are known to influence the *d-excess* of water vapor that originates over the ocean^30^. The July 2013 cyclone event led to a combination of the following changes in Arctic Ocean characteristics: 1) increased wind speeds; 2) reduced sea ice cover; 3) greater relative humidity above the ocean surface; and 4) decrease in ocean surface temperature^31^,^32^. Given that these changes are likely to result in lower *d-excess* values^16^,^26^,^33^,^34^, the sharp drop in *d-excess* during the Arctic cyclone event is consistent with expectations.

## Mechanisms responsible for the cyclone isotope excursion

**Figure 3:**
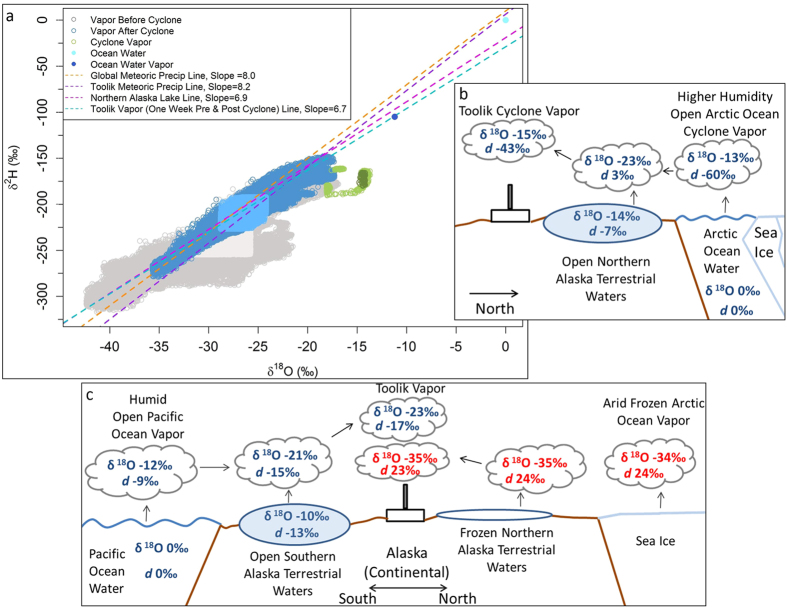
Panel a) Water vapor isotope ratio values (one second temporal resolution) from before cyclone (May 15-July 24), during cyclone on July 25 (including the transitional values around the cyclone measurements), and after cyclone (July 26 to August 20) and Arctic Alaska isotope water lines compared with the global meteoric water line. The water vapor isotope values before and after the cyclone measurements on July 25 are within the before and after data groups. Gray, blue, and green color tones near the center of each vapor category are the 50% probability distributions of values, while other points are the 99.3% probability distribution. Schematics depicting the influence of: Arctic Ocean water on the enriched δ^18^O and low *d-excess* values in Toolik water vapor during the cyclone (Panel b); and open waters on high and low *d-excess* values from the north and south of Toolik, respectively (Panel c).

Comparison of the water vapor isotope ratios from the periods before, after, and during the cyclone along a water line reveals both the δ^18^O enrichment and the uniqueness of the cyclone event (Fig. 3a). The Toolik and Global Meteoric Water Lines (for precipitation) have similar slopes of ~8, while northern Alaska lake waters have a slope of 6.9, which is indicative of evaporation^29^. Additionally, the water line for Toolik vapor one week before and after the cyclone event has a slope of 6.7, similar to that of northern Alaska lake waters. A schematic (Fig. 3b,c), in combination with the different water lines, reveals a process that could result in the anomalous δ^18^O and *d-excess* values during the cyclone. The schematic focuses on δ^18^O, as the changes to δ^18^O were greater than δ^2^H during the cyclone isotope excursion, and *d-excess*. It is assumed that air parcels precipitated moisture on their topographic ascent from the coast to Toolik. However, this general precipitation-driven depletion^29^ is not included in the schematic as it focuses on the influence of the different water source characteristics on isotopes before these source waters are exposed to relatively similar patterns of overland isotopic depletion.

First, under equilibrium conditions when Arctic Ocean water, with a δ^18^O of ~0‰ (i.e., Vienna-Standard Mean Ocean Water), evaporates, its estimated vapor δ^18^O value is approximately −13‰^29^ (Fig. 3b). As northern Alaska lake waters have δ^18^O values of approximately −14‰^35^, which deplete to ~−23‰ when evaporated^36^, the Arctic Ocean vapor parcel moving from north to south becomes depleted as it accumulates this isotopically depleted lake water evaporate. This mixing of vapor from the Arctic Ocean and northern Alaska lake water evaporation results in water vapor arriving in the Brooks Range foothills with a δ^18^O value of approximately −15‰ (Fig. 3b). On the water line, this process corresponds with a shift of the relatively enriched cyclone water vapor isotope ratio values to the right of vapor values before and after the cyclone, indicating an Arctic Ocean contribution to the water vapor source during the cyclone isotope excursion (Fig. 3a). After the cyclone water vapor passes, the water vapor isotope ratios revert back to more typical regional values, which are characterized by more continental water vapor sources.

Northern air parcels reaching the foothills of the northern Brooks Range from a frozen Arctic Ocean have a high *d-excess* value (Fig. 2), as a frozen Arctic is an arid vapor source that results in relatively depleted δ^18^O and higher *d-excess* values (Fig. 3c). Conversely, an open Arctic Ocean is a relatively humid vapor source, which has δ^18^O values more similar to air parcels with Pacific Ocean sources (compared to a closed Arctic Ocean), owing to the availability of open water (Fig. 3c). Previous studies^16^,^26^,^28^,^33^,^34^ indicate increased relative humidity over the ocean decreases *d-excess* values. Therefore, the cyclone *d-excess* values are lower than typically expected from vapor above the ocean surface because of the high humidity associated with the cyclone event. The isotope and trajectory data suggest that air parcels reaching the northern foothills of the Brooks Range are largely derived from two source groups: humid (i.e., open Arctic or Pacific Ocean), relatively enriched δ^18^O and low *d-excess* vapors, and arid (i.e., frozen Arctic Ocean), relatively depleted δ^18^O and high *d-excess* vapors (Figs. 2,3). The δ^18^O enrichment during the cyclone event indicates an open ocean water moisture source for the air parcel that reached the northern foothills of the Brooks Range, while the low *d-excess* excursion suggests unusually high humidity for this moisture source. Thus, the cyclone event provides an important opportunity to measure the water vapor isotope ratios of an unusually high humidity open Arctic Ocean environment associated with a cyclone, which has not been previously done and is critical to understanding water isotope-derived Arctic paleoclimate records. Of particular importance are paleoclimate records of warm periods in which it is hypothesized that a decrease in sea ice extent was coupled with increased atmospheric moisture from open northern oceans^22^,^23^,^37^.

## Paleoclimatic implications of cyclone isotope characteristics

Similar to the transitions observed in this 2013 cyclone, rapid decreases in *d-excess* values are also present in paleoclimate ice core records collected from the Greenland Ice Sheet. For example, cores from the Greenland ice core project (GRIP) reveal that rapid warming events such as the end of the Younger Dryas (YD), the shift to the Bolling-Allerod (BO) interstadial, and the Dansgaard-Oeschger (DO) cycles are characterized by sharp decreases in ice core *d-excess* values^23^. Additionally, the *d-excess* changes are negatively correlated with δ^18^O during these periods: *d-excess* decreases while δ^18^O increases, which is the same as the isotopic relationship recorded during the 2013 cyclone (Fig. 1). There is also consistency in these relationships between different Greenland ice cores. The Northern Greenland ice core (North GRIP) shows a similar pattern of lower *d-excess* values during warming events to the patterns from the Central Greenland (GRIP) and Southern Greenland (Dye 3) ice cores, suggesting a regional signal^22^. Further, this consistency of isotope values across different Greenland ice cores shows that the association between rapidly retreating sea ice and warmer Arctic temperatures, such as the end of the YD, is potentially recorded isotopically in ice cores as sharp decreases in *d-excess*. Previous studies hypothesized that during these periods of rapid warming and retreat of sea ice cover, such as those in the GRIP record, sharp drops in *d-excess* values were influenced by the incorporation of open north Atlantic Ocean water into the moisture source^21^,^22^. Since the heating of air increases its capacity to hold moisture^38^, a warming climate combined with an open ocean as a moisture source likely resulted in greater water vapor concentration above the northern oceans during warm periods. Similarly, as the cyclone isotope excursion is associated with increases in both open Arctic Ocean water and humidity, we postulate that the cyclone induced drop in *d-excess* is representative of past warm periods in which sea ice cover retreated and atmospheric humidity above the Arctic Ocean was greater.

The speed of these decreases in *d-excess* and how they relate to temperature changes can also vary across transitional events in the GRIP record^23^. Based on the cyclone isotope data, it is then possible that the variability in the rate of *d-excess* drops in the ice core records is influenced by the speed of sea ice retreat: a quicker retreat equates to a steeper *d-excess* decline. Further, DO events 19 and 5 (GRIP core) show a drop in *d-excess* before associated increases in temperature^23^. As the cyclone isotope data reveal a mechanistic connection between lower *d-excess* values and oceanic water moisture sources, this lag suggests that sea ice retreated prior to warming. Similarly, decreases in *d-excess* occurred quicker than corresponding warming in Greenland at both the BO transition (GRIP core)^23^ and at the end of the YD (Dye 3 core)^21^, which also suggests that increases in temperature lagged the retreat of sea ice. Although changes in temperature are the response of various different climate forcings^39^, it is possible that during some previous climatic transitions the retreat of sea ice in Earth’s northern Polar regions led to increased northern temperatures.

The decrease in ocean surface temperature during the cyclone event and its drop in *d-excess* (for more details refer to S2.2) might initially appear incongruous with a comparison to warmer periods in the Arctic paleoclimate record that also reveal sharp decreases in *d-excess*^23^. However, Greenland paleoclimate data suggest a decoupling between the cooling temperatures of the oceanic moisture source and the warming temperatures at the ice sheet depositional site during warming periods in the GRIP record^23^. It is hypothesized that this difference is due to a large change to the North Atlantic hydrologic cycle related to diminished sea ice: the retreat of sea ice during rapid warming periods exposed large areas of cold surface water that were incorporated into oceanic moisture sources^23^. Sea ice retreat and incorporation of oceanic moisture sources into the hydrologic cycle are thought to be necessary for these antiphase patterns of oceanic moisture source cooling and warming at the Greenland ice sheet site to occur. Similarly, the cyclone also caused a reduction of sea ice and churning of newly uncovered and cold Arctic Ocean water, which contributed to increased atmospheric moisture and led to the low *d-excess* values during the cyclone event we measured. Previous studies of potential proxies for past changes in sea ice lacked information about the connection of chemical markers between their oceanic source, atmospheric transfer, and deposition at ice sheets^40^. This study presents an unprecedented explanation of the processes by which higher northern ocean surface humidity (relative to average modern conditions) could result in the deposition of exceptionally low *d-excess* values in a terrestrial environment. Our Arctic cyclone isotope data link ocean and atmosphere changes in the Arctic hydrologic cycle during warm periods in the paleoclimate record and provide the first direct isotope evidence of a hypothesized sea ice retreat and increased distribution of ocean water moisture sources.

## Methods

A Picarro L2130-*i* analyzer (hereafter Picarro water vapor analyzer) measured δ^18^O, δ^2^H, and water concentration (g/kg) of vapor. The L2130-*i* is a cavity ring down-spectroscopy (CRDS) analyzer, which is based on cavity-enhanced, near-infrared laser absorption spectroscopy procedures, tuned on a narrow spectral region^41^,^42^,^43^. The Picarro water vapor analyzer, powered by a nearby solar array, was deployed in undisturbed moist acidic tundra on a small knoll about 1 km from the Toolik camp (Fig. S3). Annually, Toolik receives ~32 cm of precipitation and the average annual temperature is -7 °C^19^. A tower was set up adjacent to the Picarro analyzer to collect air samples and meteorological data. A tube was placed at the top of the tower (3.2 m) and connected to the analyzer and samples were collected from May 11, 2013 to August 20, 2013. Samples were collected through the tube with a pump, which ran continuously at a rate of 5 liters/second. The ratios of water vapor isotopes were measured approximately every second by the Picarro water vapor analyzer and five-minute average values were evaluated in this study (except Fig. 3a, which utilizes higher temporal resolution data for presentation purposes). Twice a day (approximately every 12 hours), standard waters were injected and analyzed to correct for potential drift of the instrument. Low absolute humidity values (e.g., <5 g/kg) can lead to isotopic biases of at least 5.0 and 50.0 per mil for δ^18^O and δ^2^H, respectively^44^. As absolute humidity during the cyclone event was >10 g/kg and within previously observed ranges (Fig. S2), the cyclone-related *d-excess* values were not likely influenced by humidity related isotopic biases. The results of stable isotope analyses are presented using the δ-notation reported relative to the Vienna-Standard Mean Ocean Water (VSMOW) standard, with an accuracy of ±0.2‰ for δ^18^O and ±2‰ for δ^2^H. Additionally, a remote, automated weather station (Campbell Scientific Custom with CR1000 Datalogger and various sensors) was established to collect meteorological data: temperature (thermistor) and relative humidity (capacitive sensors) at 1.7 m and wind speed and direction (Young Windbird) at a 3.2 m height. Sensors were scanned at 30 second intervals and average readings were logged every five and 30 minutes from May 11, 2013 to August 20, 2013, same as the collection time period of isotope data. The day of year (DOY) is presented in Julian form and commences at midnight in the Alaska Standard Time zone.

Deuterium excess (δD – 8*δ^18^O), or *d-excess*, values were calculated from the isotopic data as they can be indicative of differing storm tracks^45^. Further, *d-excess* depends mainly on the surface temperature and relative humidity of the moisture source, but is generally more sensitive to changes in relative humidity^16^,^26^,^27^,^28^,^33^,^34^. The *d-excess* of water vapor above the ocean is largely influenced by the following at the moisture source: composition of sea surface isotopes; sea surface temperatures; air temperature; and relative humidity^46^. As these source oceanic isotopic conditions are mostly preserved in the *d-excess* values as an air parcel moves from its source to a collection site, *d-excess* can help understand the environmental and isotopic conditions of a moisture source. Comparatively, the separate δ^2^H and δ^18^O isotopes are more influenced by local environmental conditions (e.g., temperature) at the sample collection site^23^,^46^. Therefore, *d-excess* is particularly useful for the study goal of understanding and interpreting the isotopic conditions of an Arctic Ocean cyclone. These *d-excess* values were stratified into two groups: high (>16.9‰) and low (<−9.8‰). High values were greater than two standard deviations above the mean *d-excess* value, while low values were lower than two standard deviations below the mean *d-excess* value. The *d-excess*, and correspon*d*ing δ^18^O, values in the Fig. 3b and 3c schematics for vapor from open or frozen ocean water and terrestrial water moisture sources are estimated^29^,^36^, while all other isotope values are known (including the northern and southern Alaska open terrestrial waters)^35^.

Air parcel back-trajectory analyses, of the *d-excess* values prior, during, and after the cyclone event, were calculated using the National Oceanographic and Atmospheric Administration’s Air Resource Laboratory HYbrid Single-Particle Lagrangian Integrated Trajectory (HYSPLIT) model^47^. The HYSPLIT model calculates the position of an air parcel using variables such as temperature, wind speed, pressure, and solar radiation. While models in previous studies primarily used wind data for back trajectory calculation^48^, meteorological variables like temperature are incorporated into HYSPLIT as they also influence air parcel movements, which were calculated within HYSPLIT by a Lagrangian three dimensional air parcel velocity algorithm^49^. The back trajectories were calculated for a 72-hr period before the time of the sample measurement at an altitude of 500 m above base-level. It is possible that evaporative processes beyond this 72-hr period influenced water vapor isotope ratios, but this impact is likely to be minimal as three days is generally long enough to identify moisture source characteristics^50^. An altitude of 500 m was selected to allow for air movement below the tropopause (usually above 10,000 m), but above the altitude where ground features generally could influence wind direction^51^. Back trajectory calculations were used to help associate particular isotopic values (e.g., *d-excess*) from vapor samples with air parcel movements that are indicative of Arctic cyclonic activity.

## Additional Information

**How to cite this article**: Klein, E. S. *et al.* Arctic cyclone water vapor isotopes support past sea ice retreat recorded in Greenland ice. *Sci. Rep.* **5**, 10295; doi: 10.1038/srep10295 (2015).

## Supplementary Material

Supporting Information
