# Supplementary material for: Arctic cyclone water vapor isotopes support past sea ice retreat recorded in Greenland ice
Source: Sci Rep. 2015 May 29;5:10295. doi: 10.1038/srep10295 (PMC4650601; doi:10.1038/srep10295)
Supplement: Supporting Information [file srep10295-s1.pdf]

Arctic cyclone water vapor isotopes support past sea ice retreat recorded in Greenland ice

Eric S. Klein[1*], J. E. Cherry[2], J. Young[2], D. Noone[3], A. J. Leffler[1], J. M. Welker[1]

[1]University of Alaska Anchorage, Department of Biological Sciences & Environment and Natural Resources Institute, [2]University of Alaska Fairbanks, International Arctic Research Center, [3] Atmospheric Sciences Department, Oregon State University
*esklein@uaa.alaska.edu

1 Supplementary Methods
2 Supplementary Discussion
2.1 Cyclone isotope excursion trajectory and influential factors
2.1.1 Wind Speed
2.1.2 Sea Ice Extent
2.1.3 Humidity Above the Ocean Surface
2.1.4 Ocean Surface Temperatures
2.1.5 Cyclone Influence on *d-excess* Values
2.2 Moisture sources and *d-excess* values
2.3 Comparison of cyclone *d-excess* values to other low *d-excess* periods
2.4 Potential instrumentation errors
3 Supplementary Figure Legends
4 Supplementary Tables
5 References for Supplementary Material

1 Supplementary Methods

Precipitation isotope samples

Precipitation samples were collected between early May and late August 2013 and 2012 (snow and rain) as well as during the prior winter (2012-2013) on an event basis in order to characterize the seasonal patterns of precipitation isotopes[1]. Precipitation samples were collected with a funnel and sample vial, frozen and then thawed at a later time, prior to isotopic analysis. Two ml of each sample was gathered and placed in septa capped glass vials. Isotopic analysis of precipitation samples were conducted on a Picarro liquid water isotope analyzer which operates similarly to the previously described Picarro L2130-*i* analyzer, equipped with a GC Pal autosampler. Similar to the water vapor isotopes, the results of stable isotope analyses are presented using the δ-notation reported relative to the Vienna-Standard Mean Ocean Water

(VSMOW) standard, with an accuracy of ±0.2‰ for $\delta^{18}O$ and ±2‰ for $\delta^{2}H$. Each of the samples was analyzed six times and reanalysis of the sample occurred if the standard deviation of the six replicates was greater than 0.3‰ for $\delta^{18}O$ and/or 3‰ for $\delta^{2}H$, or if the internal standard for the run differed from the accepted value by greater than ±0.2‰ or 2‰, for $\delta^{18}O$ and $\delta^{2}H$ respectively.

2 Supplementary Discussion

2.1 Cyclone isotope excursion trajectory and influential factors

The back trajectory analyses indicate that the air parcel associated with the cyclone and isotope excursion was sourced in the high Arctic and followed a trajectory that was both different than before and after the event (Fig. S1) and unique to the record (Fig. S2). Prior to the cyclone, DOY 204, air parcels were approaching Toolik Lake (Fig. S3) from the southwest and had relatively average *d-excess* values (Fig. S1). During DOY 205, the trajectories began to progressively move north into the Arctic Ocean. This northern trajectory progression continues until reaching its highest latitude around the middle of DOY 206 (purple line on Fig. S1), which coincides with the sharp decrease in *d-excess* values. Additionally, as all trajectories are estimated for 72 hours, the length of this northern most trajectory reveals that the speed of the air parcel movement increased substantially around the cyclone event and isotope excursion. Following this, near the end of DOY 206, the storm trajectories began to move south and *d-excess* values increased. This southern trajectory shift continues into DOY 207, until the trajectories at the end of DOY 208 become similar to those before the cyclone event and *d-excess* values return to near average (Fig. S1). Increased wind speeds, reduced sea ice extent, greater

humidity above the ocean surface, and lower ocean surface temperatures all influenced the low *d-excess* values associated with the cyclone.

2.1.1 Wind Speed

Firstly, analysis of data indicating sea ice speed and drift[2,3] show that during the cyclone event the speed of sea ice, along the air parcel trajectory associated with the isotope excursion, more than doubled as it increased to nearly 0.3 m/s (Fig. S4a). Previous studies have shown that sea ice motion is primarily forced by, and is linearly related to, wind speed; with sea ice drifting at about 2% of surface wind speed[4,5]. Based on this relationship, it is estimated that surface winds above the ocean during the cyclone event reached nearly 15 m/s, which is supported by atmospheric reanalysis models[6]. Wind speeds greater than 7 m/s above an ocean result in a rough evaporative environment[7], which leads to decreased *d-excess* values of moisture evaporated from the ocean[8]. Therefore, the almost 15 m/s surface winds above the Arctic Ocean during the cyclone likely led to an environment in which lower *d-excess* values would be expected. After the cyclone event passed, the sea ice speed and drift reduced and the *d-excess* values increased (Fig. S4a).

2.1.2 Sea Ice Extent

Secondly, sea ice data[2,3] indicate that the air parcel associated with the cyclone event had a trajectory that primarily passed over a retreating sea ice complex comprised of a mix of open ocean and sea ice less than 1 m thick (Fig. S4b). The increased wind speed associated with the cyclone likely hastened the retreat of sea ice cover[9]. Moreover, as greater open ocean can increase storm generated surface winds in the Arctic Ocean[10], the loss of sea ice during the cyclone event could have been a positive feedback to surface wind speeds. Our comparison of *d-*

excess and air parcel trajectories in this study found a significant correlation between low *d-excess* values and availability of open water moisture sources (Fig. 2). Therefore, the low *d-excess* values associated with the cyclone event and open ocean might be expected.

2.1.3 Humidity Above the Ocean Surface

Next, the Toolik data reveal a sharp spike in absolute humidity that coincides with the isotope excursion associated with the cyclone event (Fig. 1c). It is possible that this humidity spike was caused by a narrow band of moisture associated with the passage of a cold front. However, a satellite image (Fig. S5a) taken within minutes of when the *d-excess* excursion was recorded shows the outer edge of the cyclone at Toolik and not a narrow moisture band. This same satellite image (Fig. S5a), however, also shows a broken line of cumuliform clouds on the outer edge of the cyclone system near the vicinity of Toolik, which indicates atmospheric convection and raises the possibility that evaporation from local scattered showers influenced the *d-excess* excursion.

Although, isotopic fractionation relationships and long-term precipitation records indicate that evaporation from local precipitation did not result in the abnormally enriched isotope values recorded at Toolik during the cyclone event. The isotope fractionation from liquid to vapor is apparent in the data in the offset between overlapping vapor and precipitation measurements, with $\delta^{18}O$ of precipitation ~9.7‰ more enriched than vapor $\delta^{18}O$, on average for all measured rain events across the record (Fig. S2a). More specifically, experiments show that the temperature dependent fractionation during a phase shift of liquid to vapor at the 7.9°C average air temperature of the *d-excess* excursion (Fig. 2) results in a $\delta^{18}O$ depletion of 10.8‰[11]. Thus in order to measure $\delta^{18}O$ vapor values of ~ -15‰, as measured during the cyclone at Toolik (Fig. 2a), corresponding precipitation $\delta^{18}O$ values would need to be approximately -4.2‰. A nearly

20 year record of Toolik precipitation isotopes and temperatures[12] (Fig. S6) shows that the most enriched $\delta^{18}O$ precipitation value at any temperature is only -8.63‰ (at 18.3°C). Conversely, the -4.2‰ precipitation value needed to result in the July Arctic cyclone vapor measurement of ~ -15‰ is much closer to the average $\delta^{18}O$ of July precipitation in the southern US state of Arkansas (-3.1‰)[13]. Further, when this Toolik precipitation record is limited to a range of temperatures ±0.5°C of those recorded when the *d-excess* excursion occurred (6.7 to 9.1 °C), the maximum, minimum, and mean $\delta^{18}O$ values are -11.76‰, -22.12‰, and -17.76‰, respectively (Fig. S6). Thus, after fractionation, the average recorded precipitation $\delta^{18}O$ value from temperatures comparable to those during the cyclone event would result in vapor $\delta^{18}O$ values of ~27‰, which are closer to the more continental water vapor source values measured before and after the cyclone (Fig. 3). Additionally, precipitation $\delta^{18}O$ values from a five year (2004-2008) study in Zhigansk, Russia (a similar latitude and distance from the Arctic Ocean as Toolik) reveal maximum values of ~-10‰[14], far more depleted than the -4.2‰ required to produce vapor $\delta^{18}O$ values of ~-15‰. Overall, measured isotope vapor values and long-term Arctic precipitation data indicate that the *d-excess* excursion was not influenced by evaporation from a local precipitation event.

Additionally, if the *d-excess* excursion was the result of a narrow moisture band and cold frontal passage, then similarly low *d-excess* values would likely be expected at other times in the record. There were other absolute humidity spikes in the record potentially associated with the passage of a cold front**,** but these were not associated with a sharp drop in *d-excess*. For example, at DOY 215.5 and 199.5 there were spikes in absolute humidity (Fig. 1c), relatively similar to the spike associated with the cyclone excursion, but there are not a noticeable changes in *d-excess* (Fig. 1b). Instead the *d-excess* excursion at Toolik is only associated with passage of

the cyclone. Previous studies[7,8,15,16] indicate that an increase in relative humidity over the ocean decreases *d-excess* values. It is likely that the combination of increased wind speed and diminished sea ice cover led to greater mixing of air and water, which increased relative humidity levels, above the Arctic Ocean during the cyclone event. Moreover, the transient spike in humidity recorded in the Toolik vapor during the decrease in *d-excess* (Fig. 1c) supports this idea of raised humidity levels during the cyclone event.

2.1.4 Ocean Surface Temperatures

Lastly, data show that during the July 2013 cyclone event, sea surface temperatures in open Arctic Ocean water around the storm trajectory generally decreased ~1°C[2,3]. This is similar to results from a study that indicates the largest change in sea surface temperature caused by an Arctic storm occurs over open water. This study found that a 2008 Arctic Ocean storm, with a similar path to the July 2013 cyclone, induced mixing of the upper ocean, which caused sea surface cooling of up to 2°C along the southern Beaufort Sea[10]. Additionally, another study also shows heat loss in the upper ocean due to an August 2012 Arctic cyclone event[9]. Therefore, given the reduced ocean surface temperatures during the 2013 cyclone event and that *d-excess* of water vapor can decrease when the temperature of the ocean surface lowers[7], a decrease in Toolik water vapor *d-excess* values could be expected during the cyclone event.

2.1.5 Cyclone Influence on *d-excess* Values

Taken collectively, it appears that the combination of increased wind speeds, reduced sea ice extent, greater humidity above the ocean surface, and lower ocean surface temperatures created an environment in which *d-excess* values reduced sharply in association with the Arctic cyclone event. This relationship also suggests a potential reason why water vapor *d-excess* values did not drop later in the season when sea ice coverage decreased after the cyclone event: there were not

strong enough winds, which would create a rough evaporative environment, mix the top of the ocean and drive the ocean surface temperatures down, and increase humidity above the ocean surface. Additionally, the recorded *d-excess* excursion did not persist for the entire duration of time that the cyclone was north of Alaska because Toolik, ~180 km south of the Arctic Ocean, was on the southern edge of the cyclone (Fig. S5a) and thus only received impacts from the cyclone for a short time period. This is supported by a satellite image of the cyclone system taken at nearly the same time as the *d-excess* excursion was recorded[17] (Fig. S5a). In this image Toolik is near the outside edge of the cyclone, which then moves east through Northern Alaska, but about 15 hours after the *d-excess* excursion the outer edge of the cyclone has already passed Toolik (Fig. S5b). The fact that Toolik was near the edge of the cyclone is also supported by wind and surface pressure data[18]. Figure S7 shows regional meteorological data about 1.5 hours before the isotopic anomaly was measured. Toolik (red diamond) is about 100 km NE of Anaktuvuk Pass (blue diamond) (Fig. S7), which shows clear skies and ~2.5 m/s winds blowing from the northeast. Conversely, the Northern Alaska coast (north of Toolik) reveals cloudy skies (as seen in Fig. S5a), rain showers, and ~10.2 – 12.8 m/s winds blowing from the west (the direction of the cyclone movement). Thus, as a result of a high pressure ridge south of the lower pressure cyclone system, the northern coast winds are at least four fold greater than those just south (Fig. S7). So Toolik is south of the high and low pressure convergence near the coast, with its windier and wetter weather, but north of the calmer and drier weather around Anaktuvuk Pass. The position of Toolik between these meteorologically different (e.g., wind speed, cloud cover) high and low pressure systems indicates it is near the continuously shifting cyclonic boundary (as supported by Fig. S5a) and would likely only receive cyclone related impacts for a short time. Therefore, it would not be expected for the anomalous *d-excess* values to be present

at Toolik for the full duration that the cyclone was around Northern Alaska. If water vapor isotope ratio data were collected closer to, or in, the low pressure Arctic Ocean system (Fig. S7) it is likely the *d-excess* excursion would have persisted longer. Additionally, even though air parcels before and after the cyclone isotope measurements also exhibit Arctic Ocean trajectories (Fig. S1), as Toolik was near the cyclonic circulation boundary, it also received moisture with source characteristics more reflective of conditions outside the cyclonic wind circulation (i.e., lower humidity above the ocean surface).

2.2 Moisture sources and *d-excess* values

The relationship between air parcel back trajectories and high and low *d-excess* values suggests that low *d-excess* values are generally associated with the availability of open water moisture sources, such as oceans. Some of the trajectories associated with low *d-excess* values have a continental path and air parcels influenced by terrestrial features, but lakes and rivers were open (i.e., not frozen) when these air parcels traversed over Alaska (Fig. 3c). Further, the influence of seasonally available open water across Alaska is apparent in the comparison of the *d-excess* values and trajectories associated with DOY 143 and DOY 222 (Fig. 2). The trajectories for both of these days are similar and largely cover the same terrestrial landscape. However, the time periods for *d-excess* measurements between these two trajectories are quite different. On DOY 143, a high *d-excess* value, the interior of Alaska still had freezing temperatures and was just emerging from an abnormally cold spring[19], which did not allow for incorporation of any terrestrial open water sources into air parcels. Conversely, a similar trajectory associated with a low *d-excess* value on DOY 222 occurred near the end of summer when water bodies were unfrozen, thus open water could be incorporated into moisture sources (Fig. 2). Another

example of the influence of open water on *d-excess* is seen in the trajectories associated with DOY 137 & 138, which have low *d-excess* values and open water moisture sources (Fig. 2). Then on the next day, DOY 139, trajectories shift northward with sources out of the Arctic Ocean and *d-excess* values switch substantially from the low to high value group, indicating a change to arid moisture sources. While the trajectories on DOY 139 pass across the Arctic Ocean, it is still frozen at that time and thus open water is not available as a moisture source.

2.3 Comparison of cyclone *d-excess* values to other low *d-excess* periods

Although the cyclone event was associated with the lowest *d-excess* value of the record (~-47‰), there were other periods with low *d-excess* values. Specifically, the second lowest *d-excess* value (~ -43‰) occurred early in the record around DOY 137 (Fig. S2b). However, there are isotopic differences between these two low *d-excess* periods. The trajectories associated with the DOY 137 low *d-excess* values reveal that the air parcels moved northward from southern Alaska (Fig. 2). As previously mentioned, these low *d-excess* values are associated with open water moisture sources, but the δ^{18}O and δ^{2}H values indicate that terrestrial open water sources (e.g., lakes and rivers) had a greater influence on the water vapor isotope ratios measured at Toolik than open ocean waters. If these DOY 137 low *d-excess* values were dominated by an oceanic moisture source when they reached Toolik, then the water vapor isotope ratio values associated with them would likely be more enriched, similar to the cyclone vapor isotope process described (Fig. 3). Instead, using the δ^{18}O values as an example, the low *d-excess* values associated with DOY 137 were comprised of δ^{18}O values more depleted (~ -26‰) and similar to the average vapor δ^{18}O value before and after the cyclone (~ -26‰; Table S1), when the isotope signature was likely dominated by evaporate from terrestrial moisture sources such as lakes.

Therefore, the DOY 137 low *d-excess* values are likely the result of the moisture source mixing with evaporate from open water bodies in southern Alaska (Fig. 3c). So while the low *d-excess* values associated with DOY 137 are related to open water bodies, they are isotopically distinct from the open ocean moisture source from the cyclone.

2.4 Potential instrumentation errors

It is possible that the isotope excursion during the cyclone is related to instrumentation error, but this appears quite unlikely. In addition to the potential isotope biases related to low humidity, erroneous values can also be due to oversaturation of the analyzer from high rainfall. There were a couple periods in which high rainfall amounts led to the accumulation of liquid water in the collection tube, which reached the analyzer and resulted in clearly erroneous values (e.g., water concentrations four fold greater than the average level). For example, Toolik received a large amount of rainfall across DOY 200 and 201, which led to oversaturation of the analyzer and erroneous isotope values (Fig. 1b). The flat, nearly horizontal line visible on the isotope graphs reveals where erroneous values were removed due to oversaturation of the analyzer. As there was not any rainfall during the cyclone isotope excursion (Fig. 1b), the isotope values related to this cyclone event are not the result of rain-induced saturation of the analyzer.

3 Supplementary Figure Legends

Supplementary Figure 1: Air parcel back trajectories (main) and *d-excess* values (inset) around the time of the Arctic cyclone. Shorter and more southern trajectories (with *d-excess* values near the mean) occur before the cyclonic event (dashed lines), while during the cyclonic event (solid lines) trajectories sweep in from the high Arctic. The cyclonic trajectory represented with the solid purple line corresponds with the lowest recorded *d-excess* values. After the cyclonic event passes, the *d-excess* values return near the mean and the back trajectories are southern again (hashed and checkered lines). Estimated sea ice extent on DOY 206 (the day of the cyclone event) is shown in dark blue. Figure created using ArcGIS 10.3.

Supplementary Figure 2: Isotope and weather data from Toolik. Panel a: Water vapor (lines) and periodic precipitation (points) isotope ratio ($\delta^{18}O$ and $\delta^{2}H$) measurements. Panel b: *d-excess* values calculated from isotopic data (for both vapor and precipitation samples). Different excursions in *d-excess* values are apparent, the largest of which (-47 ‰) occurred on DOY 206. Precipitation values are also presented. Panel c: Air temperature and absolute humidity. Below freezing temperatures and snow events were recorded at both the start and the end of the season. Panel d: Wind speed and direction. Direction is dominated by northern (~360/0°) and southern (~180°) winds.

Supplementary Figure 3: Study site location of Toolik Field Station in Arctic Alaska. Toolik is in the northern foothills of the Brooks Range about 185 km south of the Arctic Ocean at an elevation of about 760 meters. Inset: The instrument tower looking south toward the Brooks Range in July (photo: ESK). Figure created using ArcGIS 10.3.

Supplementary Figure 4: Arctic Ocean sea ice speed (Panel a) and sea ice thickness (Panel b) before (DOY 204), during (DOY 205 & 206), and after (DOY 207) the cyclone event. The isotope excursion was measured at Toolik on DOY 206. During the cyclone, sea ice speed more than doubled in many parts of the Arctic Ocean (Panel a). The trajectory associated with the isotope excursion primarily passed through open water and retreating sea ice (red oval, Panel b). Figure modified from Navy Coupled Ocean Data Assimilation figures[3].

Supplementary Figure 5: Satellite images (visible sensor) showing Toolik (red point) relative to the cyclone at two different time periods. Panel a: Photo taken about 6 minutes (11:22 AM AST) before the *d-excess* excursion was recorded at Toolik on DOY 206 (July 25). Note that Toolik is near the outer edge of the cyclone, visible primarily in the Arctic Ocean to the north. Panel b: Photo taken about 16 hours after the *d-excess* excursion was recorded (3:57 AM AST on DOY 207). Note that the cyclone has moved east and past Toolik. Images modified from NOAA satellite images[17] .

Supplementary Figure 6: Precipitation $\delta^{18}O$ and air temperature relationships for Toolik from samples collected between 1995 and 2013. The red diamonds represent 254 samples collected at Toolik, while the open blue circles indicate the samples with a range of temperatures ±0.5°C of those recorded when the cyclone *d-excess* excursion occurred (6.7 to 9.1 °C). The horizontal blue line indicates the approximate $\delta^{18}O$ value, based on estimated temperature dependent fractionation[11], needed to result in the vapor $\delta^{18}O$ value measured during the cyclone excursion, which is much closer to the mean $\delta^{18}O$ value of July precipitation in the southern US state of Arkansas (-3.1‰)[13] than any of the Toolik measurements.

Supplementary Figure 7: Surface pressure map of Northern Alaska and the Arctic Ocean ~1.5 hours before the isotope excursion was measured. Toolik (red diamond) is between high and low pressure systems, and their corresponding differences in variables such as wind speed and cloud cover, and close to the continuously shifting cyclone boundary (Fig. S5a) defined by these pressure differences. Figure modified from NOAA Weather Prediction Center Surface Analysis Archive[18].

4 Supplementary Tables

Supplementary Table 1: Water vapor isotope values ($\delta^{18}O$, $\delta^{2}H$, *d-excess*): during the cyclone event and isotope excursion; one week before and after the cyclone (including the values from the cyclone); and the overall record. Water vapor isotope values during the cyclone event were different than isotope values before and after the cyclone. The most enriched $\delta^{18}O$ values and lowest *d-excess* values of the overall record occurred during the cyclone event.

Time period	$\delta^{18}O$ (‰)		δD (‰)		d-excess (‰)	
	Range	Mean	Range	Mean	Range	Mean
DOY 206.42 - 206.44 (cyclone event only)	-19.5 to -15.8	-17.6	-187.8 to -174.2	-181.0	-31.0 to -47.8	-39.4
DOY 199 - 214 (one week before and after the cyclone event)	-30.8 to -15.8	-25.6	-241.2 to -157.6	-200.4	-47.8 to 21.6	4.31
DOY 136 - 230 (overall record)	-42.6 to -15.8	-28.2	-308.3 to -149.0	-222.8	-47.8 to 42.9	2.96

5 References for Supplementary Material

1 Welker, J. Isotopic (δ18O) characteristics of weekly precipitation collected across the USA: an initial analysis with application to water source studies. *Hydrological Processes* **14**, 1449-1464 (2000).
2 Cummings, J. A. Operational multivariate ocean data assimilation. *Quarterly Journal of the Royal Meteorological Society* **131**, 3583-3604, doi:10.1256/qj.05.105 (2005).
3 NavalResearchLaboratory. *Real-time 1/12° Arctic Cap HYCOM/CICE/NCODA Nowcast/Forecast System (ACNFS) with NAVGEM atmospheric forcing* <http://www7320.nrlssc.navy.mil/hycomARC/arctic.html> (2014, Date of access 15/02/2014).
4 Leppäranta, M. *The drift of sea ice*. (Springer, 2005).
5 Thorndike, A. & Colony, R. Sea ice motion in response to geostrophic winds. *Journal of Geophysical Research: Oceans (1978–2012)* **87**, 5845-5852 (1982).
6 NOAA. *6-Hourly NCEP/NCAR Reanalysis Data Composites*, <http://www.esrl.noaa.gov/psd/data/composites/hour/> (2014, Date of access: 20/08/2014).
7 Jouzel, J. *et al.* Water isotopes as tools to document oceanic sources of precipitation. *Water Resources Research* **49**, 7469-7486, doi:10.1002/2013WR013508 (2013).
8 Benetti, M. *et al.* Deuterium excess in marine water vapor: Dependency on relative humidity and surface wind speed during evaporation. *Journal of Geophysical Research: Atmospheres* **119**, 584-593, doi:10.1002/2013JD020535 (2014).
9 Zhang, J., Lindsay, R., Schweiger, A. & Steele, M. The impact of an intense summer cyclone on 2012 Arctic sea ice retreat. *Geophysical Research Letters* **40**, 720-726, doi:10.1002/grl.50190 (2013).
10 Long, Z. & Perrie, W. Air-sea interactions during an Arctic storm. *Journal of Geophysical Research: Atmospheres (1984–2012)* **117**, doi:10.1029/2011JD016985 (2012).
11 Majoube, M. Fractionnement en oxygene-18 et en deuterium entre l'eau et sa vapeur. *Journal of Chemical Physics* **197**, 1423-1436 (1971).
12 Klein, E. *et al.* In review. McCall Glacier record of Arctic climate change: Interpreting a Northern Alaska ice core with regional precipitation and water vapor isotopes. *Quaternary Science Reviews* (2014).
13 Welker, J. M. ENSO effects on δ18O, δ2H and d-excess values in precipitation across the US using a high-density, long-term network (USNIP). *Rapid Communications in Mass Spectrometry* **26**, 1893-1898 (2012).
14 Kurita, N. Origin of Arctic water vapor during the ice-growth season. *Geophysical Research Letters* **38**, doi:10.1029/2010GL046064 (2011).
15 Johnsen, S., Dansgaard, W. & White, J. The origin of Arctic precipitation under present and glacial conditions. *Tellus B* **41**, 452-468, doi:10.1111/j.1600-0889.1989.tb00321.x (1989).
16 Merlivat, L. & Jouzel, J. Global climatic interpretation of the deuterium-oxygen 18 relationship for precipitation. *Journal of Geophysical Research: Oceans (1978–2012)* **84**, 5029-5033, doi:10.1029/JC084iC08p05029 (1979).
17 NOAA. (ed Department of Defense Air Force Space and Missile Center) (National Geophysical Data Center's Solar Terrestrial Physics Division Earth Observation Group, 2013).

18 NOAA. *Weather Prediction Center: Surface Analysis Archive*, <http://www.hpc.ncep.noaa.gov/archives/web_pages/sfc/sfc_archive_maps.php?arcdate=07/25/2013&selmap=2013072518&maptype=lrgnamsfc> (2013, Date of access: 11/11/2014).

19 ACRC. in *Climatological Data: Monthly Time Series* Vol. 2014 (Alaska Climate Research Center, Anchorage, 2014).

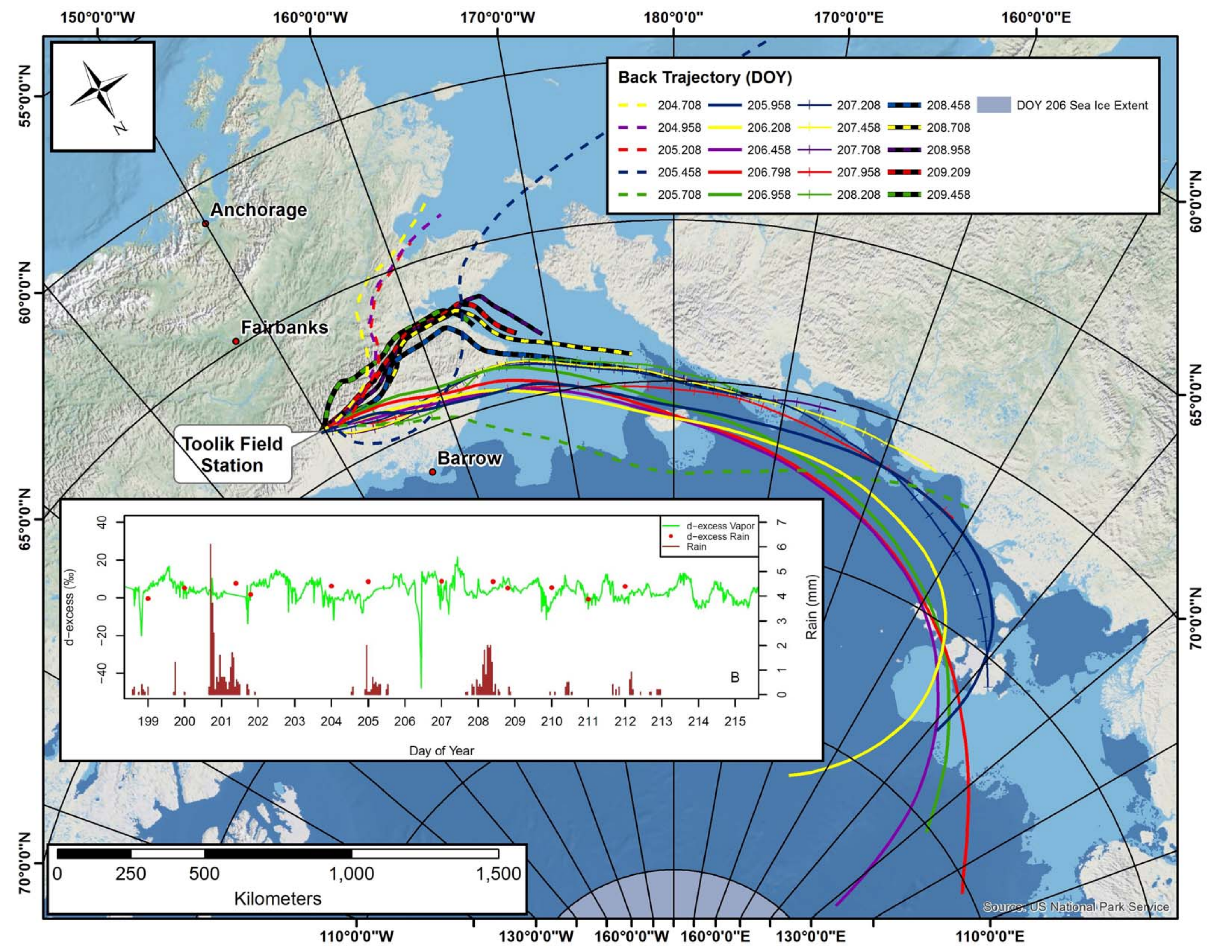

150°0'0"W
160°0'0"W
170°0'0"W
180°0'0"
170°0'0"E
160°0'0"E
55°0'0"N
60°0'0"N
65°0'0"N
70°0'0"N
N
Back Trajectory (DOY)
204.708
204.958
205.208
205.458
205.708
205.958
206.208
206.458
206.798
206.958
207.208
207.458
207.708
207.958
208.208
208.458
208.708
208.958
209.209
209.458
DOY 206 Sea Ice Extent
Anchorage
Fairbanks
Toolik Field Station
Barrow
d−excess Vapor
d−excess Rain
Rain
d−excess (‰)
Rain (mm)
Day of Year
B
0
250
500
1,000
1,500
Kilometers
110°0'0"W
130°0'0"W
160°0'0"W
160°0'0"E
130°0'0"E
110°0'0"E
Source: US National Park Service

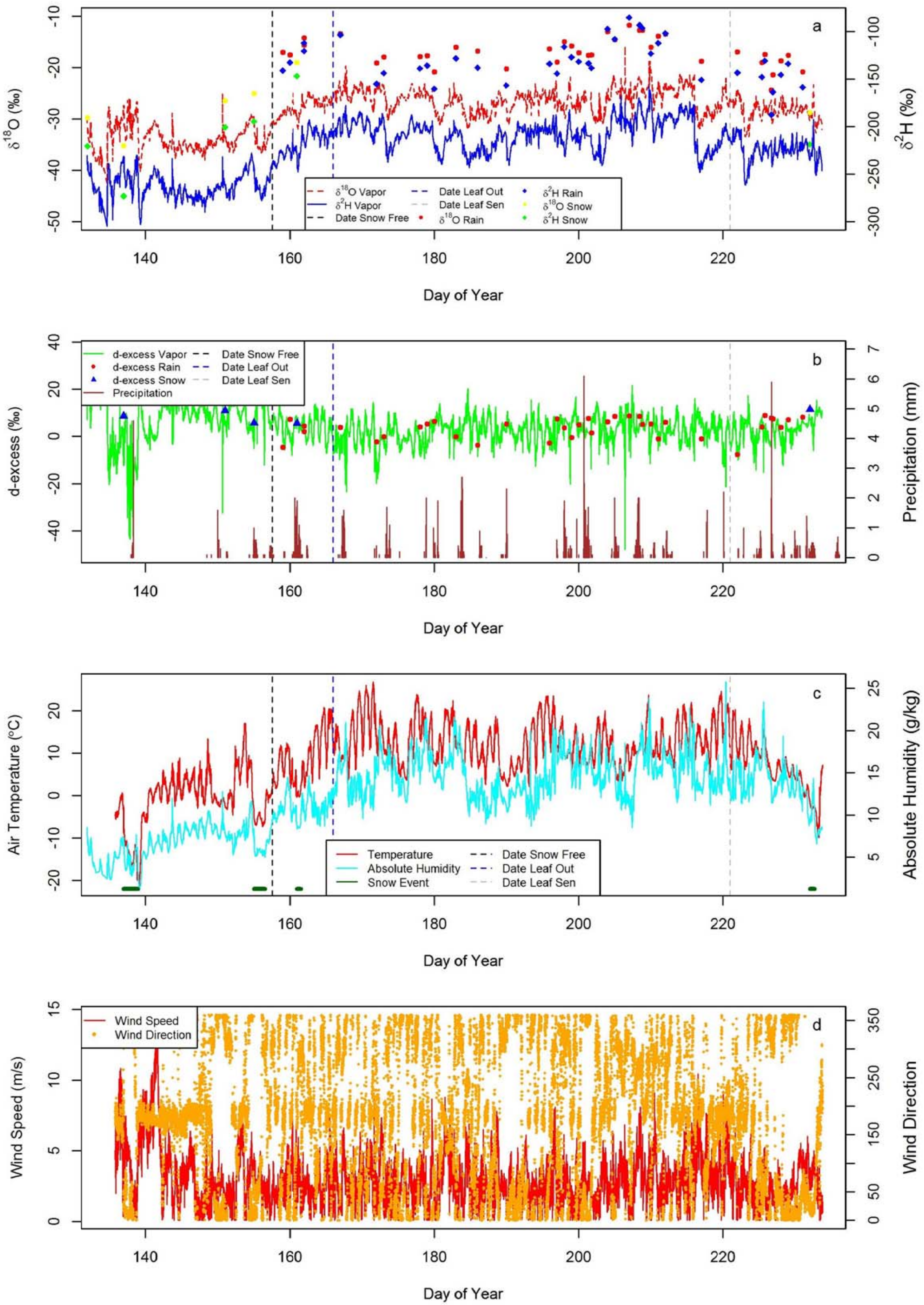
a
δ18O (‰)
δ2H (‰)
δ18O Vapor
δ2H Vapor
Date Snow Free
Date Leaf Out
Date Leaf Sen
δ18O Rain
δ2H Rain
δ18O Snow
δ2H Snow
Day of Year
b
d-excess (‰)
Precipitation (mm)
d-excess Vapor
d-excess Rain
d-excess Snow
Precipitation
Date Snow Free
Date Leaf Out
Date Leaf Sen
Day of Year
c
Air Temperature (°C)
Absolute Humidity (g/kg)
Temperature
Absolute Humidity
Snow Event
Date Snow Free
Date Leaf Out
Date Leaf Sen
Day of Year
d
Wind Speed (m/s)
Wind Direction
Wind Speed
Wind Direction
Day of Year

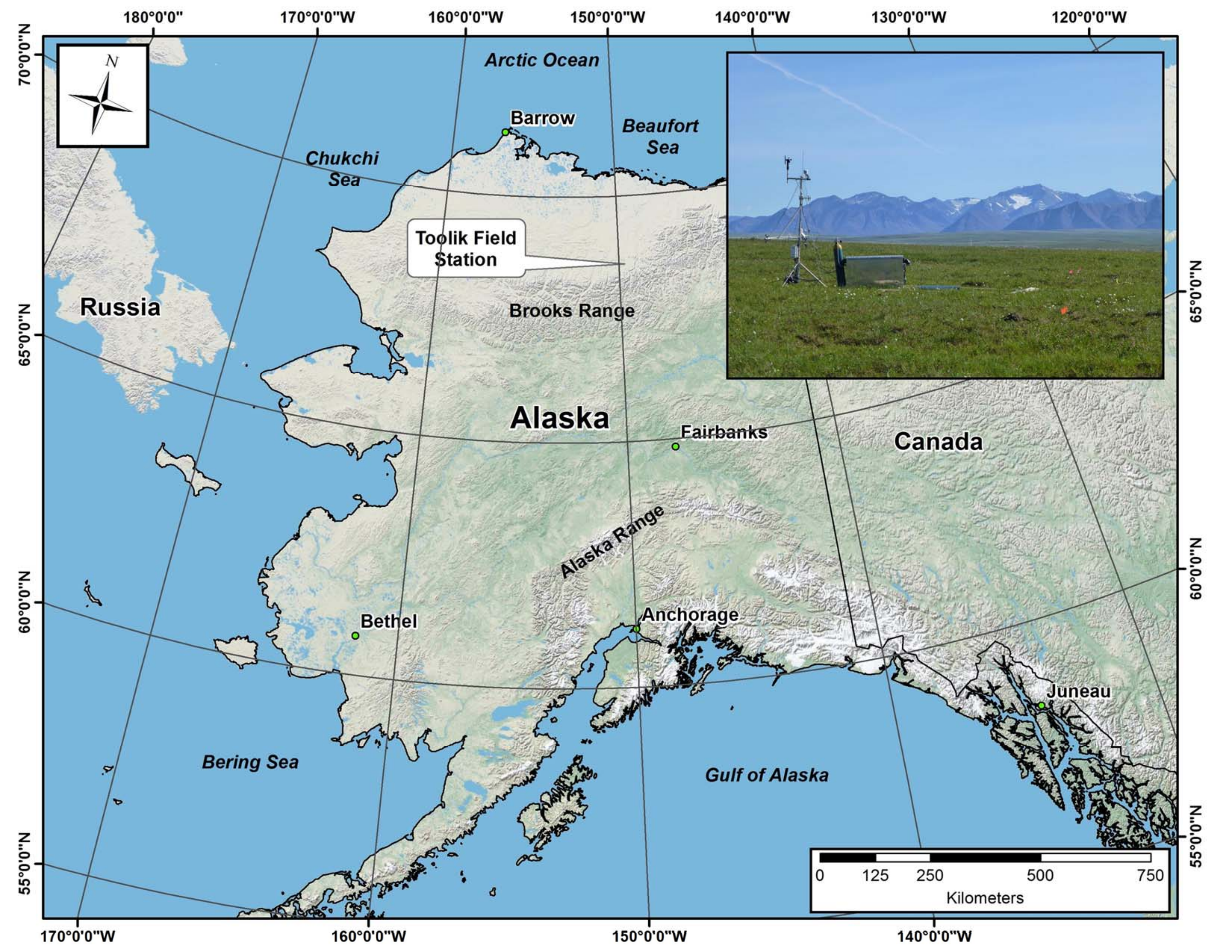

180°0'0"
170°0'0"W
160°0'0"W
150°0'0"W
140°0'0"W
130°0'0"W
120°0'0"W
70°0'0"N
65°0'0"N
60°0'0"N
55°0'0"N
N
Arctic Ocean
Barrow
Beaufort Sea
Chukchi Sea
Toolik Field Station
Russia
Brooks Range
Alaska
Fairbanks
Canada
Alaska Range
Anchorage
Bethel
Juneau
Bering Sea
Gulf of Alaska
0 125 250 500 750
Kilometers

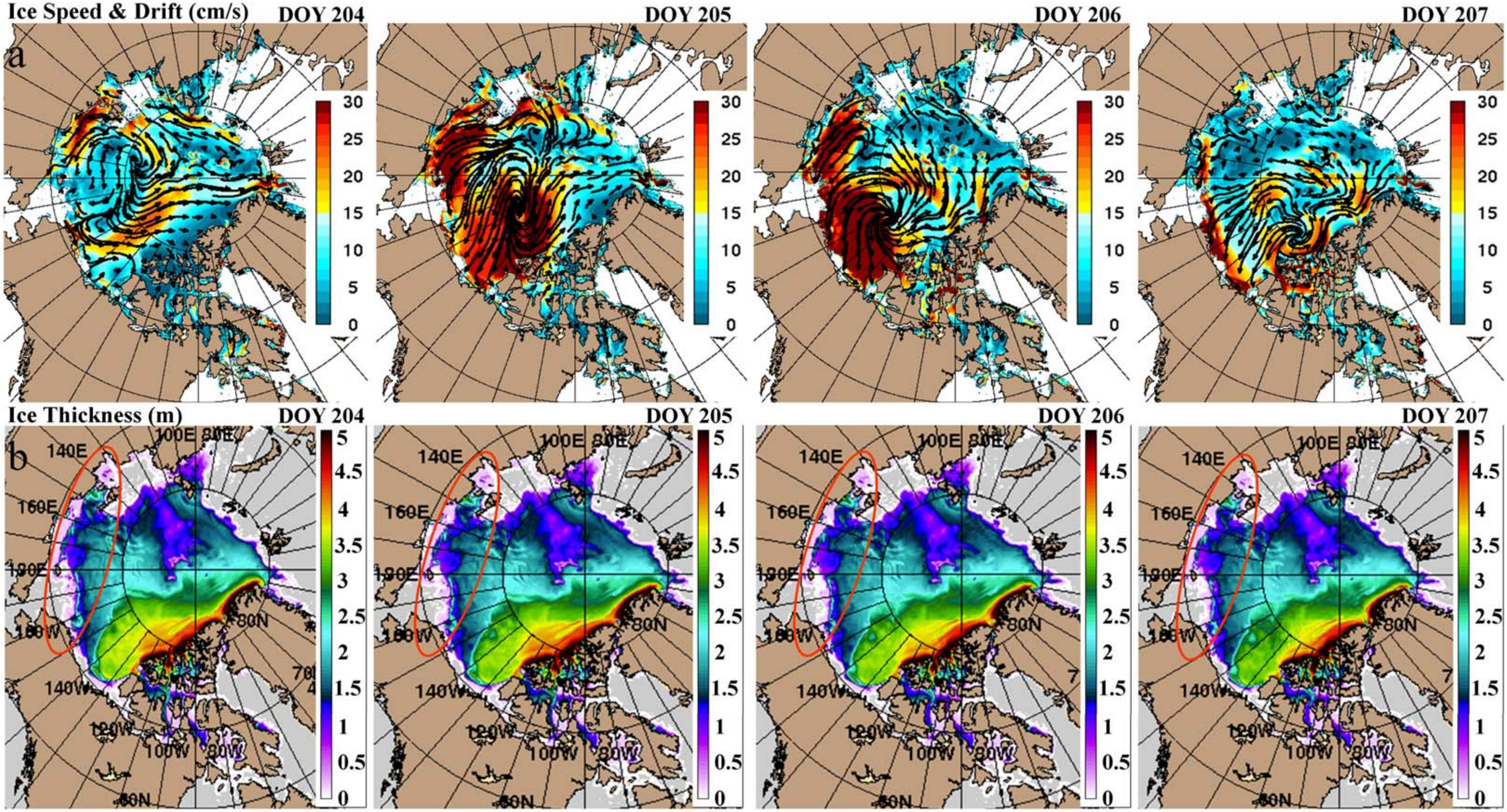

Ice Speed & Drift (cm/s)
DOY 204
DOY 205
DOY 206
DOY 207
a
30
25
20
15
10
5
0
Ice Thickness (m)
DOY 204
DOY 205
DOY 206
DOY 207
b
140E
160E
100E
80E
80N
140W
120W
100W
80W
5
4.5
4
3.5
3
2.5
2
1.5
1
0.5
0

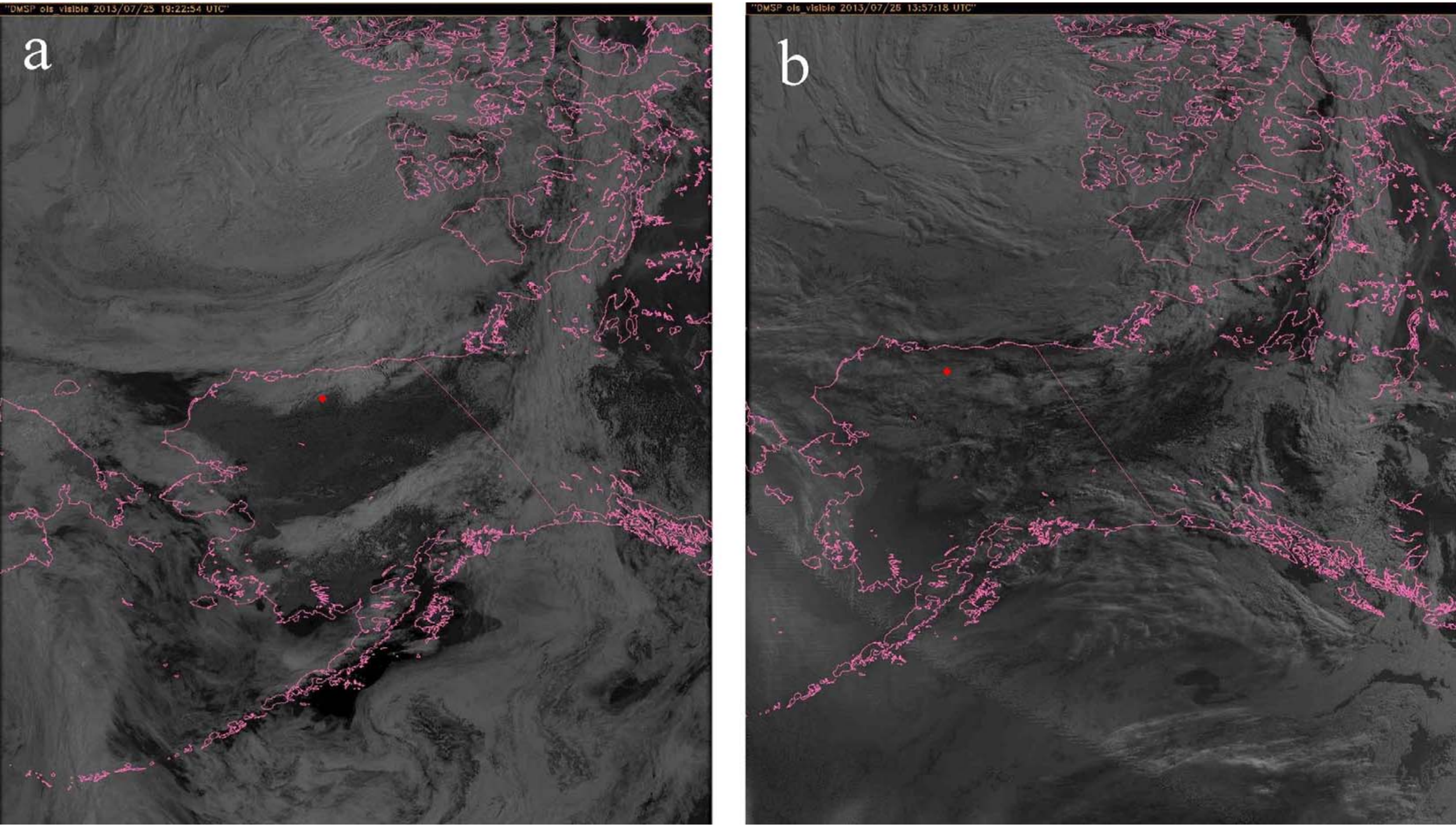
"DMSP ols_visible 2013/07/25 19:22:54 UTC"
a
"DMSP ols_visible 2013/07/25 13:57:18 UTC"
b

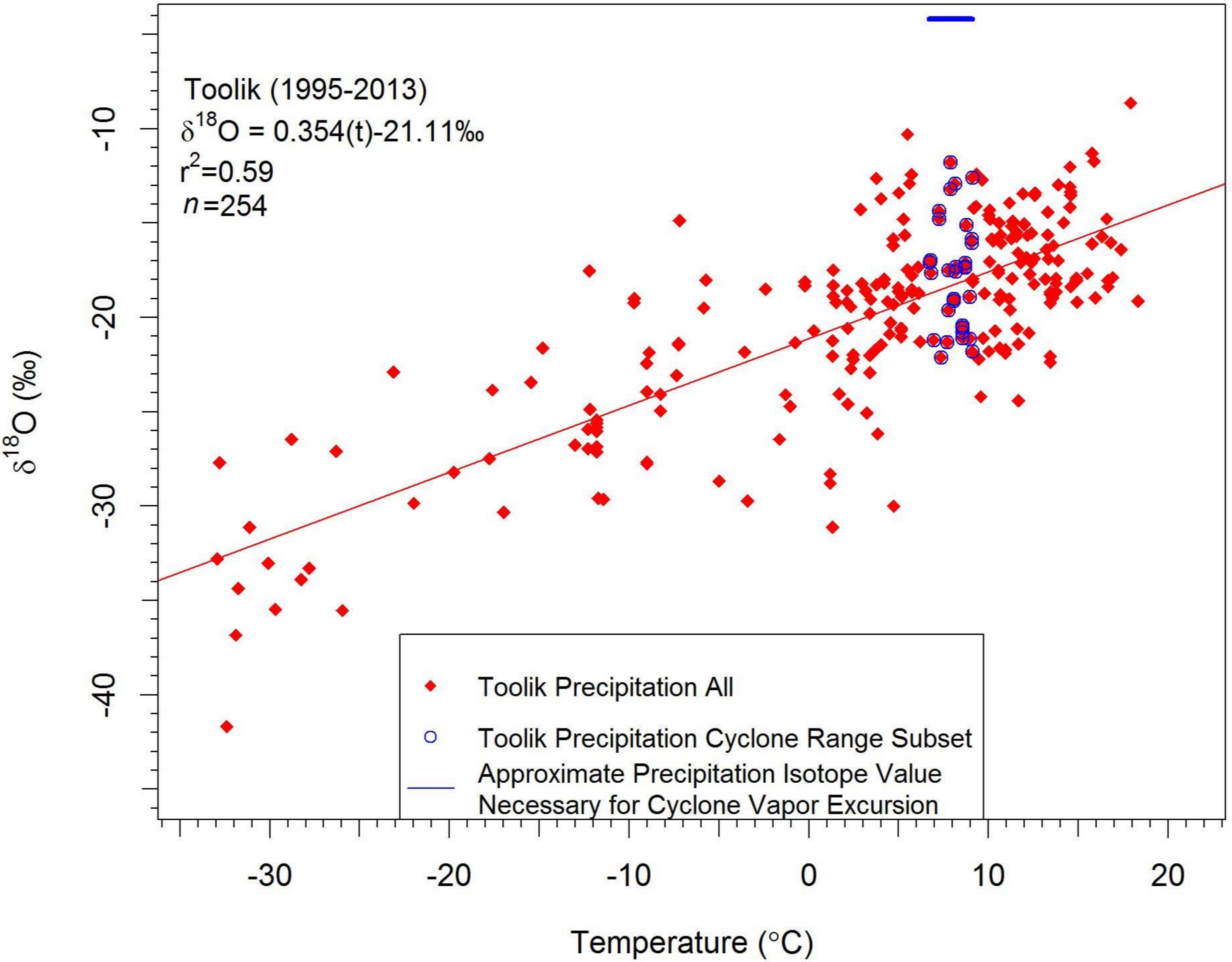
Toolik (1995-2013)
δ18O = 0.354(t)-21.11‰
r2=0.59
n=254
-10
-20
-30
-40
δ18O (‰)
-30
-20
-10
0
10
20
Temperature (°C)
Toolik Precipitation All
Toolik Precipitation Cyclone Range Subset
Approximate Precipitation Isotope Value Necessary for Cyclone Vapor Excursion

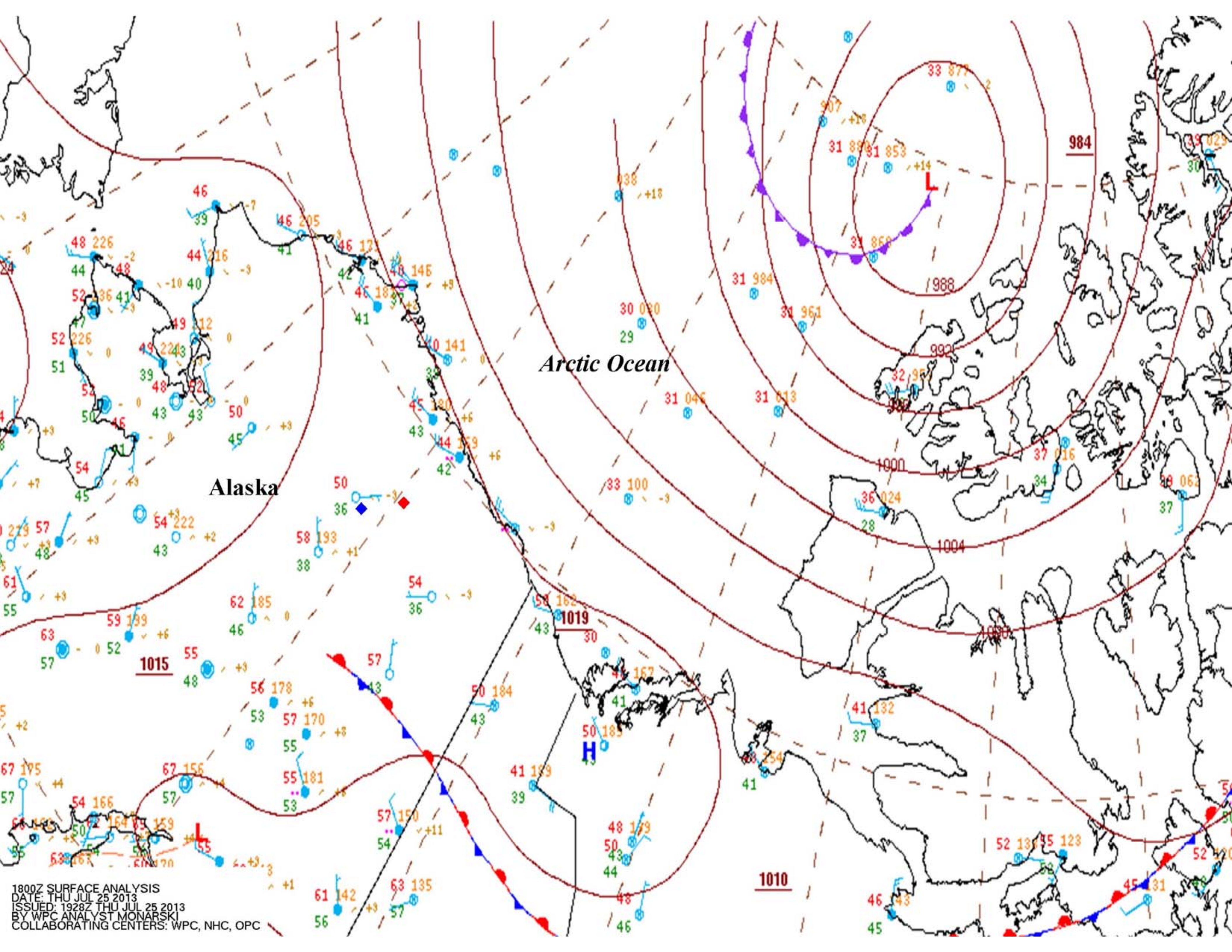
Arctic Ocean
Alaska
984
988
992
1000
1004
1010
1015
1019
1010
1800Z SURFACE ANALYSIS
DATE: THU JUL 25 2013
ISSUED: 1928Z THU JUL 25 2013
BY WPC ANALYST MONARSKI
COLLABORATING CENTERS: WPC, NHC, OPC
